# Genomic medicine goes mainstream

**DOI:** 10.1038/npjgenmed.2015.1

**Published:** 2016-01-13

**Authors:** Stephen W Scherer

**Affiliations:** 1 The Centre for Applied Genomics, The Hospital for Sick Children, University of Toronto, Toronto, ON, Canada; 2 McLaughlin Centre, University of Toronto, Toronto, ON, Canada

I believe that we will look back upon 2015 as the year when whole-genome sequencing (WGS) went mainstream, and the concept of *genomic medicine* started to move into broad practical application. Each human genome is a blueprint, sculpted by millennia of natural selection, and individualised through meiosis. Its decoding will enable formulated health-related decisions that will become an increasing part of our daily existence.

*npj Genomic Medicine* is a new international, peer-reviewed journal dedicated to publishing the most important scientific advances in genomics as applied to the practice of medicine. As its inaugural editor, my working definition of genomic medicine is as follows: diagnosis, prediction, prognosis, prevention and/or treatment of disease and disorders of the mind and body, using approaches informed or enabled by knowledge of the genome and the molecules it encodes.^1^ Our goal is to publish outstanding papers that describe genome-related studies of individuals, families or populations in a medical context. We will emphasise the coupling of detailed phenotype and genome sequence, genotype, or mutational information in delineating the underlying aetiology of disease.

The journal will also welcome detailed case or family reports where *n*=1. I can see a time in the near future where such studies of individuals carrying presumed highly penetrant mutations but who are ‘resilient’ for that particular disorder will rival the number of papers describing patients who do succumb to their genetic liability. We will also encourage sharing of ideas, as well as recommendations and/or guidelines of how such data should be used in the clinical management of study subjects and others.

Crystal gazing, based on my own experiences and those of our distinguished Associate Editors, I foresee that in the journal’s first few years, the most impactful areas we hope to publish on will include the following (a few recent studies from the editorial group’s own work are cited for reference): (1) WGS in clinical diagnosis and prognosis;^2–7^ (2) WGS applied to non-invasive prenatal diagnosis;^8,9^ (3) WGS characterisation of the ~99% of the genome not readily captured through less comprehensive technologies^10,11^, (4) discovery of new regulatory variants related to risk of disease;^12,13^ (5) new approaches including computational algorithms, population genetics and/or functional experimentation for interpreting data;^14–20^ (6) databases to allow universal access of massive genomic data sets for research;^21^ and (7) all of the above integrated and interfaced with ‘smart phone’ technology and other ‘big data’ innovation, which is part of our inevitable future.

As genomic information may inform on past, present and future outcomes of a personal and sensitive nature, it warrants handling with thought and care. We will encourage papers in areas of ethics, health economics and law, as they apply to medical genomics and influence our rapidly changing society.^22–25^ In my own research area of autism genomics, I am increasingly enthralled how cultural evolution driven by the information era may be influencing our natural evolution, and what the consequences are for medicine in the twenty-first century^26^.

To capture all of these advances, the journal will publish Articles, Case or Resource Reports, Brief Communications, Commentaries and Review Articles. A professionally written Editorial Summary will accompany each Article, summarising the key issues addressed. The speed of moving papers through the peer-review system will be paramount to our success, but only those papers deemed outstanding and strategic to the journal’s mission will be published. With extensive support from *Nature*, the material published in *npj Genomic Medicine* will also be disseminated broadly via social media.

The journal recognises the importance of the deposition of published data in the appropriate accessible databases and it will follow existing *Nature* guidelines to ensure these standards are upheld. The way these often large and complex data sets are handled, stored, shared and analysed is evolving, falling into the realm of what is now called ‘big science’, something the journal will also cover.

The impact of genomics is now ubiquitous in health research, both basic and clinical, and must be supported by impactful worldwide journal representation. We will wholeheartedly strive to have as many members from as many different countries and backgrounds involved in the different aspects of journal. Diversity drives our genetic history and we believe it is also crucial to the journal’s success. In fact, in agreeing to accept the role of editor, one of my main motivations was to try to use genomic medicine as the *medium* ^26^ to deliver the message of science to continue to build a global village striving for the optimal health of all of its inhabitants.
